# Chronic effects of a static stretching intervention program on range of motion and tissue hardness in older adults

**DOI:** 10.3389/fmed.2024.1505775

**Published:** 2024-11-25

**Authors:** Masatoshi Nakamura, Antonino Scardina, Ewan Thomas, Konstantin Warneke, Andreas Konrad

**Affiliations:** ^1^Faculty of Rehabilitation Sciences, Nishi Kyushu University, Saga, Japan; ^2^Sport and Exercise Sciences Research Unit, Department of Psychology, Educational Science and Human Movement, University of Palermo, Palermo, Italy; ^3^Institute of Psychology, Leuphana University Lüneburg, Lüneburg, Germany; ^4^Institute of Human Movement Science, Sport and Health, University of Graz, Graz, Austria

**Keywords:** ankle dorsiflexion, flexibility, stretch tolerance, passive stiffness, passive stretching

## Abstract

**Introduction:**

Clinically, knowing whether a static stretching (SS) intervention program conducted for several weeks can reduce passive muscle stiffness is important. Still, only a few previous studies have evaluated the chronic effects of an SS intervention program in older adults, and the potential relationship between ROM changes and muscle stiffness changes is still unclear. This study aimed to investigate the effects of a 10- week SS intervention partially supervised program on joint range of motion (ROM) and tissue hardness in older adults.

**Methods:**

The SS intervention program was conducted at least three times a week for 10 weeks in the ankle plantar flexor muscles of 24 community-dwelling older adults (73.8 ± 5.1 years; height: 156.0 ± 6.8 cm; body mass: 52.7 ± 8.0 kg). The SS intervention program consisted of 4 × 30-s repetitions. Ankle joint dorsiflexion (DF) ROM and tissue hardness of the medial gastrocnemius were measured before and after the 10-week SS intervention program.

**Results and discussion:**

The results showed that the 10-week SS intervention program significantly increased DF ROM (+9°, *p* < 0.01, Cohen’s *d* = 1.37) and decreased tissue hardness (−0.9, *p* = 0.04, Cohen’s *d* = −0.27). However, there was no significant correlation between these changes (*r* = 0.086, *p* = 0.561). The results of this study suggest that a 10-week SS intervention program can effectively increase DF ROM and decrease tissue hardness but that the increase in DF ROM is related to stretch tolerance rather than changes in tissue hardness.

## Introduction

Previous studies have shown that static stretching (SS) intervention programs can increase range of motion (ROM) (1–3) and improve gait function (3). ROM is generally known to decline in older populations (2, 4, 5). This age-related decline in ROM leads to decreased mobility and balance function (6) and an increased risk of falls (7). Thus, ROM-enhancing methods such as SS interventions could likely be beneficial for increasing ROM and improving health in older populations.

Several studies have suggested that resistance training can increase joint ROM (8–10). Recently, a systematic review and meta-analysis by Alizadeh et al. (11) reported that long-term resistance training increases joint ROM, and this increase has the same effect size as stretch training. In particular, resistance training emphasizing eccentric contraction results in a large increase in ROM (12). According to this evidence, resistance training and SS training programs can be useful intervention methods when the goal is to increase the ROM of a joint. Although very high-volume SS training programs (e.g., 1 h per day) can increase muscle strength and hypertrophy (13, 14), resistance training can be considered the most time-efficient method if the goal is to increase muscle strength and muscle hypertrophy.

On the other hand, one of the advantages of an SS intervention program is the reduction of passive muscle stiffness (15, 16). Interestingly, an increase in antagonist passive muscle stiffness inhibits the primary active muscle movement, resulting in high energy and metabolic costs (17, 18). Therefore, decreasing passive muscle stiffness following an SS intervention program for several weeks could be essential for maintaining physical function in older adults. In a previous study examining the acute effects of SS in older adults, Nakamura et al. (2) showed that a single 300-s bout of an SS intervention can decrease the stiffness of the medial gastrocnemius (MG) and lateral gastrocnemius muscles. In addition, a systematic review and meta-analysis by Nakamura et al. showed that a single bout of an SS intervention can decrease passive muscle stiffness in older adults to a small magnitude, and the effects are comparable between older and young adults (19). Clinically, knowing whether an SS intervention program conducted for several weeks can reduce passive muscle stiffness is important. Still, to the best of our knowledge, only a few previous studies have evaluated the chronic effects of an SS intervention program in older adults (3, 20). Although SS intervention programs appear to be effective in increasing ROM in the older adult population (21, 22), no evidence is available for potential changes in muscle stiffness. Therefore, there is a need to revisit the chronic effects of SS intervention programs on passive muscle stiffness in older adults and the potential relationship between ROM changes and muscle stiffness changes.

Thus, this study aimed to investigate the effects of a 10-week SS intervention program on ankle dorsiflexion (DF) ROM and tissue hardness in community-dwelling older adults. In previous studies, it was found that an SS intervention program can effectively reduce passive muscle stiffness in young adults (23), and a single bout of an SS intervention can effectively reduce passive muscle stiffness in older adults to a similar extent as in young adults (19). Thus, we hypothesized that the SS intervention program would effectively increase ROM and reduce tissue hardness (an index of passive muscle stiffness) in older adults. Moreover, we also hypothesized that the changes in ROM would be correlated with the changes in muscle stiffness.

## Methods

### Experimental design

**Figure 1:**
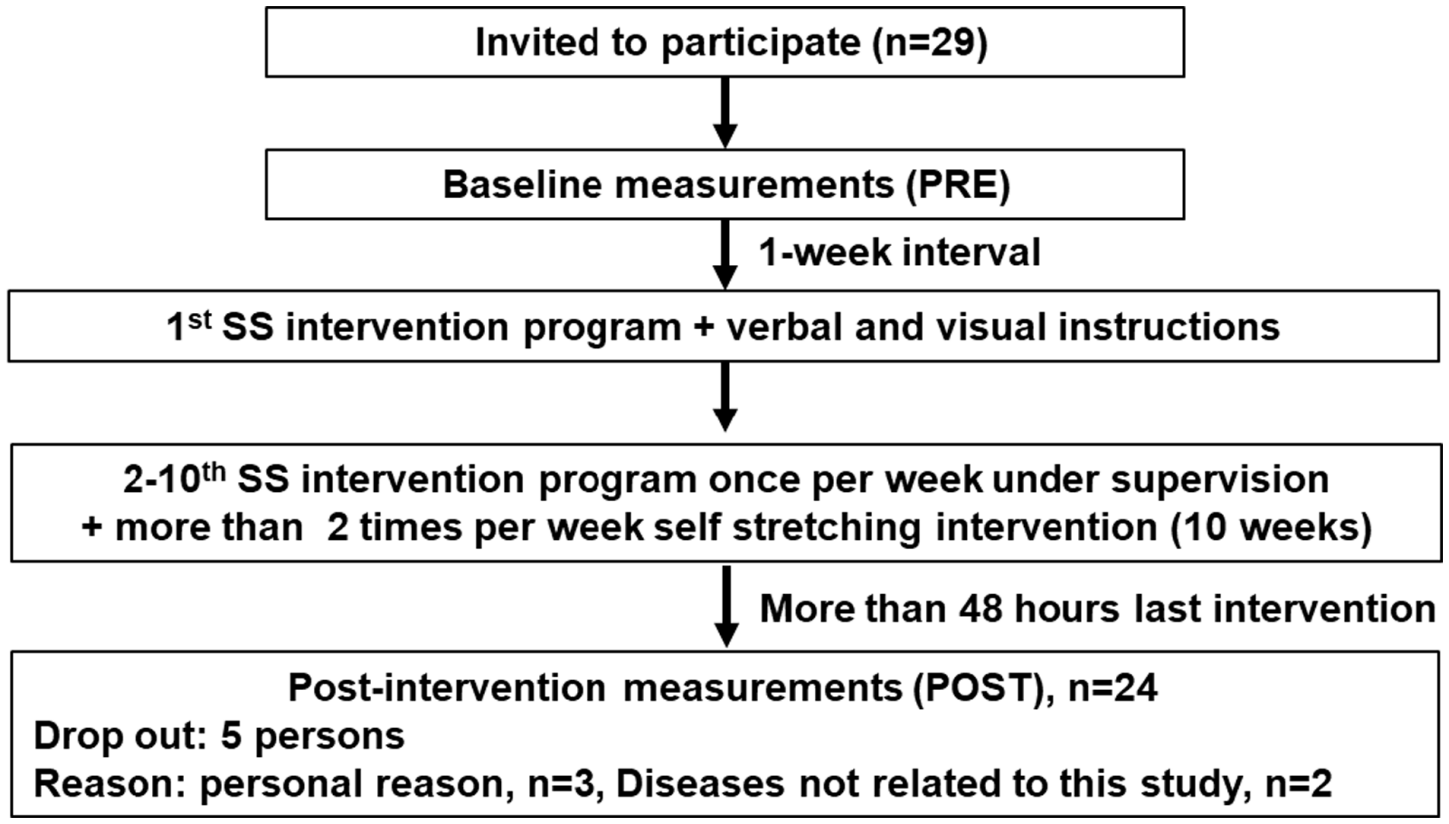
Experimental flow of this study.

In this study, participants visited the laboratory 12 times, for two measurement sessions and 10 SS intervention sessions (Figure 1). DF ROM and tissue hardness measurements in both the right and left sides were taken at the first visit (PRE measurement). The next 10 visits were made every week, and stretching of the ankle plantar flexor muscle group was performed under the supervision of the same instructor for 4 × 30-s repetitions. The SS intervention program was first performed on the right side, followed by SS on the left leg. In addition, the SS intervention program was performed more than twice per week in the home for 10 weeks. Adherence throughout the intervention program was monitored through weekly written journals, and the journal of each participant was checked every week. The last visit (12th visit) to the laboratory was conducted 48 h after the last SS intervention. Because all the participants in this study performed the SS intervention program, due to ethical considerations, there was no control group. The study was approved by the Ethics Committee of Nishi Kyushu University (R5-08) and performed in accordance with the Declaration of Helsinki.

### Participants

The DF ROM and tissue hardness of the MG in both legs were measured in 29 older adults (male, *n* = 7; female, *n* = 22). Using flyers, we recruited older adults who were participating in health classes for older adults held by the university. The inclusion criteria were age > 60 years, living in the community, and being able to walk independently. Exclusion criteria were cognitive impairment, severe cardiac or musculoskeletal disorders, previous diagnosis of pulmonary disease, and hearing impairment. Five participants dropped out because of personal reasons (*n* = 3) and diseases not related to this study (*n* = 2). Thus, the total number of participants included in the analyses of the present study was 29, and their ages ranged from 62 to 85 years (average ± SD; age: 73.8 ± 5.1 years; height: 156.0 ± 6.8 cm; body mass: 52.7 ± 8.0 kg).

In this study, we used G* power 3.1 software (Heinrich Heine University, Düsseldorf, Germany) for the sample size calculation for a paired *t*-test (effect size = 0.80 [large], *α* error = 0.05, and power = 0.95), based on the result of a previous study on the change in DF ROM after an SS intervention program (3). Thus, more than 23 participants were needed in this study. All the participants were fully informed of the purpose and procedure of the study. All participants gave written informed consent prior to participation.

### Outcome assessment

#### Assessment of DF ROM

The subject was supine with the knee joint in full extension (i.e., anatomical position). We measured the ankle DF ROM twice in 1° increments using a plastic goniometer, and the mean value at each measurement period was used for further analysis. The DF ROM was defined as the ankle joint angle just before the participant started to feel discomfort or pain (24–26). Using PRE data, we calculated the intraclass correlation coefficient (ICC) to check the test–retest reliability. The ICC of the DF ROM was 0.925 (95% confidence interval (CI): 0.82–0.968).

#### Assessment of MG tissue hardness

MG tissue hardness was measured using a portable tissue hardness meter (NEUTONE TDM-N1; TRY-ALL Corp., Chiba, Japan). The participant was instructed to lie supine on the treatment table. The hip and knee joints were set at 0°, and the participant was instructed to relax the ankle joint in a fully plantar flexed position. Tissue hardness was measured at the proximal 30% of the lower leg length (from the popliteal crease to the lateral malleolus) (27, 28). The tissue hardness meter measured the pushing force until a 14.71 N (1.5 kgf) pressure was reached (29–31). The participant was instructed to relax while the tissue hardness was measured three times at each measurement, and the mean value at each measurement period was used for further analysis. The ICC of the tissue hardness was 0.981 (95% CI: 0.962–0.991).

#### SS intervention program

Four repetitions of a 30-s self-administered ankle dorsiflexion SS intervention were performed once per week in a laboratory under direct supervision and more than twice per week in the home for 10 weeks. SS was performed with a straight knee joint to target gastrocnemius stretching. The participant was instructed to place the stretching leg as far posteriorly as possible while pushing the heel down to the ground, with the forefoot pointing forward until the point of discomfort. The SS intervention was first performed on the right leg, followed by the left leg.

### Statistical analysis

The statistical analysis was performed using SPSS (version 28.0; SPSS Japan Inc., Tokyo, Japan). The Shapiro–Wilk test was used to test the normality of the data. The PRE and POST values for each variable followed normality. Therefore, we compared the PRE and POST values using the paired t-tests. Effect sizes are reported with Cohen’s *d* and categorized as either a small effect (*d* < 0.5), medium effect (*d* = 0.5–0.8), or large effect (*d* > 0.8) (32). In addition, changes from PRE to POST values were calculated, and the Shapiro–Wilk test showed that these data were normally distributed. Thus, the relationship between the changes in DF ROM and the change in tissue hardness was examined using the Pearson product–moment correlation coefficient. A *p-*value of < 0.05 indicates statistical significance.

## Results

**Figure 2:**
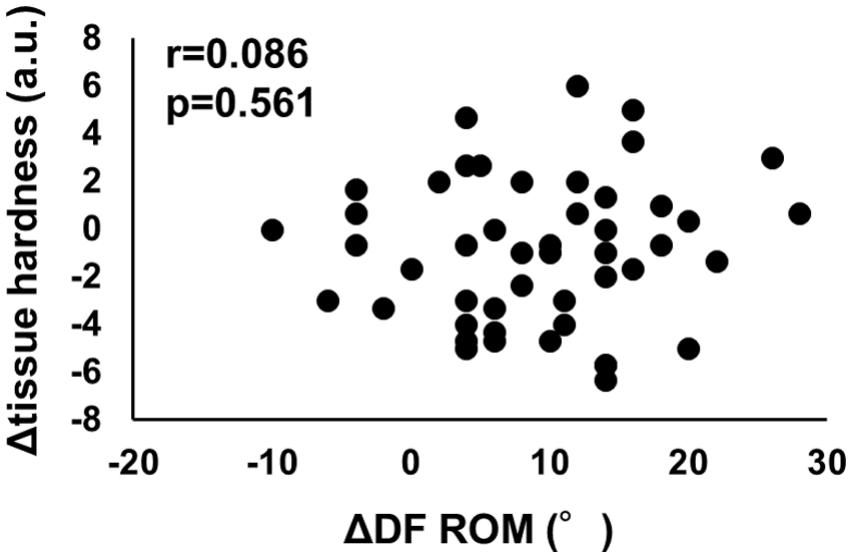
Relationship between change in dorsiflexion range of motion (DF ROM) and change in tissue hardness before and after the 10-week SS intervention program.

Table 1 lists the results for the DF ROM and tissue hardness before and after the intervention. DF ROM showed a significant increase (*p* < 0.01, *d* = 1.37, 95% CI = −11.5 to −6.77), and tissue hardness showed a significant decrease (*p* = 0.042, *d* = −0.27, 95% CI = 0.036 to 1.81). However, there was no significant correlation between the change in DF ROM and tissue hardness (Figure 2, *r* = 0.086, *p* = 0.561).

**Table 1:** Changes in dorsiflexion range of motion (DF ROM) and tissue hardness before (PRE) and after the 10-week SS intervention program (POST).

	PRE	POST	*p*-value	Effect size	95% confidence interval
DF ROM (°)	14.6 ± 6.1	23.8 ± 7.2	*p* < 0.01	*d* = 1.37	−11.5 to −0.68
Tissue hardness (a.u.)	19.2 ± 3.5	18.3 ± 3.3	*p* = 0.042	*d* = −0.27	0.04 to 1.81

## Discussion

In this study, we investigated the chronic effects of a 10-week SS intervention program on DF ROM and tissue hardness of the MG in older adults. The results showed that the 10-week SS intervention program increased DF ROM and decreased tissue hardness, but the change in tissue hardness was small (Cohen’s *d* = −0.27), while DF ROM showed a large change (Cohen’s *d* = 1.37). Since there was no significant correlation between the change in DF ROM and tissue hardness (Figure 2), the increase in ROM in the SS intervention program was not related to a change in muscle stiffness but to a change in sensation, i.e., stretch tolerance.

Our results showed that a 10-week SS intervention program can increase DF ROM in older adults, which was consistent with the findings of previous studies (3, 20). In addition, a 10-week SS training program can also decrease the tissue hardness of the MG in older adults. However, the mechanism of the decrease in tissue hardness after a 10-week SS intervention program is unclear. Nevertheless, previous studies have pointed out that changes in the flexibility of the connective tissue surrounding muscle fibers can be considered to influence decreased muscle stiffness (33, 34). Furthermore, a recent systematic review and meta-analysis concluded that SS intervention programs can increase fascicle length at rest and during stretching in healthy young populations (35). Thus, it will be necessary to investigate the mechanism of the change in muscle stiffness during an SS intervention program in older adults by conducting a detailed study that includes measurement of changes in fascicle length.

Mechanical and sensory theories have been proposed as mechanisms for the ROM increase after SS intervention programs (36). As described above, the change in tissue hardness was small (Cohen’s *d* = −0.27), while DF ROM showed a large change (Cohen’s *d* = 1.37). Furthermore, there was no significant correlation between the change in DF ROM and tissue hardness (Figure 2). These results suggest that sensory theory is associated with the increased ROM seen in this SS intervention program in older adults. Previous studies have also suggested that sensory theory is related to the increase in ROM in SS intervention programs for young adults (25, 26). These results indicate that ROM increases after SS intervention programs in young and older adults and that this increase is related to changes in stretch tolerance, i.e., sensory modification, rather than changes in muscle stiffness. Interestingly, it has been reported that there is a time gap between ROM changes and muscle stiffness changes during an SS intervention program. That is, changes in ROM have been reported to occur earlier than decreases in muscle stiffness (37). Therefore, the decrease in muscle stiffness may occur later than the change in ROM in an SS intervention program in older adults. Therefore, when an SS intervention program is designed to reduce muscle stiffness, it is necessary to measure not only the change in ROM but also muscle stiffness to monitor whether or not there is a decrease in muscle stiffness.

In this study, we focused on a specific SS training protocol to ensure consistency and observe specific effects on flexibility and ROM in a controlled manner. However, research suggests that other techniques, such as dynamic stretching or proprioceptive neuromuscular facilitation (PNF), can also increase ROM. A systematic review with meta-analyses that investigated the chronic effect of different stretching techniques on ROM showed a significant difference between the stretching techniques, indicating that SS and PNF stretching intervention programs produced greater enhanced ROM than ballistic/dynamic stretching intervention programs (22). However, a previous study comparing the effects of a single bout of SS exercise and PNF stretching exercise on older adults reported that while the increase in ROM was similar, only SS exercise effectively decreased muscle stiffness (38). Therefore, SS and PNF stretching intervention programs are effective when the goal is to increase ROM in older adults. Still, SS intervention program might be adopted when the goal is to reduce muscle stiffness. In addition, the previous study has shown that the effect of increasing ROM in a stretching intervention program is not affected by the difference in the stretched muscles (22). Therefore, although this study targeted the plantar flexor muscles of the ankle joint, it is possible to expand the results to other muscles. In the future, conducting a detailed study of the effects of different stretching techniques on ROM and muscle stiffness in various muscles will be necessary.

There are several limitations to this study. Firstly, there was no control group in this study. Therefore, it is unclear whether the changes seen in this study originated from the SS intervention program. However, this study also showed high measurement reproducibility (26, 33). Therefore, the changes in ROM and tissue hardness seen in this study were likely due to the SS intervention program. Second, we did not measure stretch tolerance or neurophysiological indices in this study. Interestingly, previous studies have reported that SS intervention programs can decrease arterial stiffness (39) and blood glucose (40). Therefore, it is necessary to comprehensively examine the effects of SS intervention programs on older adults. Third, the sample size calculation in this study was based on the effect size of dorsiflexion ROM improvements observed in the previous study. Consequently, this study might lack sufficient statistical power to detect significant effects in tissue hardness measurements or perform robust correlation analyses. Future studies with a larger sample size are recommended to confirm the findings related to tissue hardness and correlation analyses more conclusively. Fourth, the method of measuring the DF ROM of the ankle joint in this study was performed in the non-weight-bearing position. Previous studies reported the usefulness of measuring DF ROM of the ankle joint in the knee extension and flexion in weight-bearing positions (41, 42). In addition, SS intervention was conducted in a weight-bearing position in this study. Therefore, in future studies, it is necessary to conduct DF ROM measurements in weight-bearing and non-weight-bearing positions and investigate the relationship with the SS intervention program method.

## Conclusion

Our results showed that a 10-week SS intervention program can increase DF ROM and decrease tissue hardness. Our results also suggested that the increase in DF ROM after an SS intervention program can be attributed to the change in stretch tolerance, which supports the sensory theory.

## Data Availability

The raw data supporting the conclusions of this article will be made available by the authors, without undue reservation.
